# Oxidative stress induced Interleukin-32 mRNA expression in human bronchial epithelial cells

**DOI:** 10.1186/1465-9921-13-19

**Published:** 2012-03-14

**Authors:** Megumi Kudo, Emiko Ogawa, Daisuke Kinose, Akane Haruna, Tamaki Takahashi, Naoya Tanabe, Satoshi Marumo, Yuma Hoshino, Toyohiro Hirai, Hiroaki Sakai, Shigeo Muro, Hiroshi Date, Michiaki Mishima

**Affiliations:** 1 Department of Respiratory Medicine, Kyoto University Graduate School of Medicine, Kyoto, Japan; 2 Health Administration Center and Division of Respiratory Medicine, Department of Medicine, Shiga University of Medical Science, Otsu, Shiga, Japan; 3 Department of thoracic surgery, Graduate School of Medicine, Kyoto University, Kyoto, Japan; 4 Health Administration Center, Shiga University of Medical Science, Seta-Tsukinowa-cho, Otsu, Shiga 520-2192, Japan

**Keywords:** COPD, acute exacerbation, IFNγ

## Abstract

**Background:**

Chronic obstructive pulmonary disease (COPD) is characterized by airflow obstruction and persistent inflammation in the airways and lung parenchyma. Oxidative stress contributes to the pathogenesis of COPD. Interleukin **(**IL)-32 expression has been reported to increase in the lung tissue of patients with COPD. Here, we show that IFNγ upregulated IL-32 expression and that oxidative stress augmented IFNγ-induced-IL-32 expression in airway epithelial cells. We further investigated transcriptional regulation responsible for IFNγ induced IL-32 expression in human airway epithelial cells.

**Methods:**

Human bronchial epithelial (HBE) cells were stimulated with H_2_O_2 _and IFNγ, and IL-32 expression was evaluated. The cell viability was confirmed by MTT assay. The intracellular signaling pathways regulating IL-32 expression were investigated by examining the regulatory effects of MAPK inhibitors and JAK inhibitor after treatment with H_2_O_2 _and IFNγ, and by using a ChIP assay to identify transcription factors (i.e. c-Jun, CREB) binding to the IL-32 promoter. Promoter activity assays were conducted after mutations were introduced into binding sites of c-Jun and CREB in the IL-32 promoter. IL-32 expression was also examined in HBE cells in which the expression of either c-Jun or CREB was knocked out by siRNA of indicated transcription factors.

**Results:**

There were no significant differences of cell viability among groups. After stimulation with H_2_O_2 _or IFNγ for 48 hours, IL-32 expression in HBE cells was increased by IFNγ and synergistically upregulated by the addition of H_2_O_2_. The H_2_O_2 _augmented IFNγ induced IL-32 mRNA expression was suppressed by a JNK inhibitor, but not by MEK inhibitor, p38 inhibitor, and JAK inhibitor I. Significant binding of c-Jun and CREB to the IL-32 promoter was observed in the IFNγ + H_2_O_2 _stimulated HBE cells. Introducing mutations into the c-Jun/CREB binding sites in the IL-32 promoter prominently suppressed its transcriptional activity. Further, knocking down CREB expression by siRNA resulted in significant suppression of IL-32 induction by IFNγ and H_2_O_2 _in HBE cells.

**Conclusion:**

IL-32 expression in airway epithelium may be augmented by inflammation and oxidative stress, which may occur in COPD acute exacerbation. c-Jun and CREB are key transcriptional factors in IFNγ and H_2_O_2 _induced IL-32 expression.

## Background

Chronic obstructive pulmonary disease (COPD) is characterized by non-fully reversible airflow obstruction and persistent inflammation in the airways and lung parenchyma [1-3]. Airway epithelial cells are one of the most important sources of inflammatory mediators that play important roles in the pathogenesis of COPD. Several reports have indicated that various factors such as smoking, infection, and proteases activate airway epithelial cells in COPD patients [2,4-6] and this is followed by the secretion of chemokines (CCL2, CXCL5, and CXCL10), inflammatory cytokines (TNFα, IL (interleukin)-7 family members (TSLP), and IL-12), and growth factors (GM-CSF and TGF-β) [1,7,8] by these activated cells.

Cigarette smoke contains oxidants and free radicals, many of which remain in the airway for long periods [9,10], is a major source of the oxidative stress that contributes to the pathogenesis of COPD. Also, activated inflammatory cells such as alveolar macrophages, neutrophils, eosinophils, monocytes, lymphocytes, and epithelial cells in the airways of COPD patients generate reactive oxidant species in response to inflammatory mediators [10].

IL-32, which was originally reported as natural killer cell transcript 4, is known to be expressed in mononuclear cells, T cells, epithelial cells, and endothelial cells in human tissues [11-14]. Cytokines such as IFNγ and TNFα promote IL-32 expression in these cells [15]. Lipopolysaccharide (LPS) also upregulates IL-32 expression in human mononuclear cells [16]. On the other hand, IL-32 induces monocytes and macrophages to produce inflammatory cytokines including TNFα, IL-1β, IL-6, IL-4, MIP, IFNγ, and IL-8 and also regulates T cell apoptosis and monocyte differentiation to macrophages [16-18].

IL-32 has also been reported to be associated with the pathogenesis of inflammatory bowel disease and rheumatoid arthritis [12,14]. Recently, it was reported that the expression of IL-32 was increased in macrophages and airway epithelial cells in the lung tissues of COPD patients compared to that in the lungs of control smokers and non-smoking subjects. Furthermore, this IL-32 expression was reported to be negatively correlated with FEV1 and positively correlated with the expression levels of TNFα and CD8 [19]. However, the mechanisms of IL-32 gene regulation in the lungs of COPD patients are still unknown.

In this study, we evaluated whether oxidative stress affects IL-32 expression induced by IFNγ and determined a mechanism by which this expression is regulated using a human bronchial epithelial (HBE) cell culture system.

## Methods

### Reagents

IFNγ was obtained from Peprotech (Rocky Hill, NJ, USA). The JNK inhibitor SP600125 was from SABioscience (Frederick MD, USA), the MEK1 inhibitor PD98059 was purchased from Invitrogen (Carlsbad, CA, USA), and the p38 inhibitor SB203580 was from Enzo Life Sciences (Plymouth meeting, PA, USA). JAK inhibitor SC204021 was from Santa Cruz Biotechnology (Santa Cruz, CA, USA). The antibodies used were as follows: anti-human IL-32 monoclonal antibody, #KU32-52, was from Biolegend (San Diego, CA, USA); anti-β-actin polyclonal antibody was from Imgenex (San Diego, CA, USA); anti-c-Jun antibody and anti-CREB antibody were from Cell Signaling Technology (Danvers, MA, USA); and anti-RNA polymerase II antibody was from Santa Cruz Biotechnology (Santa Cruz). CREB and c-Jun siRNA were from Cell Signaling Technology. Control siRNA was from Invitrogen.

### Human bronchial epithelial (HBE) cells

HBE cells were provided from the Lung Registry of Kyoto University. The Kyoto University review board for human studies approved the protocols employed in this study, and written informed consent was obtained from all patients. Briefly, HBE cells were isolated from patients who underwent lung lobectomy for tumor resection at Kyoto University Hospital and cultured in LHC-9 medium, (Invitrogen, Carlsbad, CA, USA). The patients were all never smokers with normal pulmonary function and had no history of pulmonary diseases. The cells were isolated from the portions of bronchus that were not involved in tumor according to a modified version of a previous method [20].

### Cell culture

All experiments were carried out using cells at passages 1 to 4. To get rid of corticosteroid from the media, HBE cells were cultured in corticosteroid-free Bronchial/Tracheal Epithelial Cell Basal Medium with growth factors (BEGM) (Lonza, Basel, Switzerland) instead of using LHC-9 for 24 hours prior to IFNγ stimulation. For H_2_O_2 _treatment, the indicated concentration of H_2_O_2 _was added to the medium 2 hours before stimulation with 10 ng/mL IFNγ. To determine the effect of modulating MAPK activity on H_2_O_2 _augmented IFNγ induced IL-32 mRNA expression, 10 μM of JNK inhibitor, 20 μM of MEK inhibitor, 10 μM of p38 inhibitor, 5 μM JAK inhibitor I, or DMSO as the vehicle were added to the cell culture media 2 hours before IFNγ stimulation. In all the treatments, the final concentration of DMSO was less than 0.075%.

### MTT cell growth assay

After stimulation with 250 μM H_2_O_2_, 500 μM H_2_O_2_, 10 ng/ml IFNγ or 250 μM H_2_O_2 _and 10 ng/ml IFNγ for 48 hours, the viability of the cells were examined using MTT cell growth assay kit (Millipore, Billerica, MA, USA) according to the manufacturer's instruction.

### RNA isolation and quantitative real-time PCR

RNA was extracted from the HBE cells after H_2_O_2 _treatment plus 4, 8, and 24 hours of IFNγ stimulation using TRIzol reagent^® ^(Invitrogen) according to the manufacturer's protocol. Quantitative real-time PCR was carried out to determine IL-32 gene expression and β-actin gene expression as an internal control using the ABI-PRISM7300 Sequence-Detection-System (Applied Biosystems). The PCR primers used to detect all splice variants [21] of IL-32 mRNA and the β-actin mRNA were as follows: forward primer for IL-32, 5'-ATC CTC AAC ATC CGG GAC AG-3'; reverse primer for IL-32, 5'-ATG AGG AGC AGC ACC CAG A-3'; forward primer for β-actin, 5'-CCG ATC CAC ACG GAG TAC TTG-3; reverse primer for β-actin, 5'-CCG ATC CAC ACG GAG TAC TTG-3'.

### Protein extraction and Western blot analysis

HBE cell lysate protein was extracted after H_2_O_2 _treatment plus 48 hours IFNγ stimulation. HBE cells were lysed in RIPA buffer (50 mM Tris HCl, 150 mM NaCl, 1% TritonX) together with protease inhibitor cocktail (SIGMA, St. Louis, MO, USA). Twenty μg of proteins were electrophoresed on SDS/15% polyacrylamide gels and transferred to nitrocellulose membranes. IL-32 and β-actin were detected using specific antibodies.

### Chromatin immunoprecipitation assays

Chromatin immunoprecipitation assays were performed using the Low Cell ChIP kit^® ^(Nippongene, Tokyo, Japan) according to the manufacturer's protocol. Briefly, HBE cells were treated with or without H_2_O_2 _and/or IFNγ for 30 minutes, and then the cells were fixed with formaldehyde to cross-link proteins with DNA. The reaction was quenched by 5 minutes treatment with 1.25 M glycine. Then, the cells were lysed, and chromatin molecules were sonicated to a length of between 200 and 1000 bps. The sonicated chromatin was incubated overnight at 4°C with magnetic bead-bound antibodies against c-Jun, CREB, RNA polymerase II or normal rabbit IgG as a negative control. After being washed, the immunoprecipitated chromatins were reverse cross-linked, and the recovered DNA was purified for real-time PCR. The PCR primers used to amplify the c-Jun and CREB binding sites in the IL-32 promoter region, which are located between nucleotides -96 to 134, relative to the transcription start site at +1, were designed according to the NM_ 001012631 NCBI sequence. The PCR primers used were as follows: the forward primer, 5'-CAA GGA CAG GGT CCA AAT TC-3', and the reverse primer, 5'-GGT CCG TCC CTG GCT GGG C-3',

### Mutagenesis of the IL-32 promoter sequence and luciferase reporter assays

We generated an IL-32 promoter construct with the sequence between nucleotides -120 and +530 relative to the transcription start site at +1 of the IL-32 gene containing the c-Jun/CREB consensus sequence by PCR using human genomic DNA as a template. The PCR primers used were as follows: the forward primer, 5'-TGA TCC AGA AGT TTC TCT GGC CTC TGG A-3', and the reverse primer, 5'-GCA GCC TCT CAC TCA CCT TCG-3', and a 4 bp mutation described in a previous report [22] was introduced into the c-Jun/CREB consensus sequence. Briefly, the TGACGTCA sequence which contains the tgacgtca c-Jun and tggctgacgtcacctt CREB consensus binding sequences and which is located from nucleotides -30 to -23 in the IL-32 gene promoter was changed to TcaatTCA using PCR primers that included these changes. Promoter constructs with mutated or respective wild-type sequences were ligated into the cloning vector and amplified in *E.coli*. As the cloned promoter sequences (with or without the mutation) were located between XhoI and HindIII restriction sites, the restriction enzyme (XhoI and HindIII) -treated promoter sequences were ligated with firefly luciferase coding reporter vectors (pGL4.10[luc2], Promega, Madison WI, USA) and referred to as pWild-Luc (without the mutation) and pMutant-Luc (with the mutation), respectively. All constructs were verified by sequencing.

To determine the transcriptional activity of these IL-32 promoter constructs, HBE cells plated at 0.5 × 10^5 ^cells/well in 12-well plates were transiently transfected with 1 μg of either pWild-Luc or pMutant-Luc per well using PrimeFect II (Takara, Otsu, Japan) according to the manufacturer's protocol. The cells were then co-transfected with 0.5 μg/well of the Renilla luciferase reporter construct (pGL4.74[hRluc/TK], Promega) to normalize for differences in transfection efficiency between wells. After 2 hours of transfection, the medium was replaced with corticosteroid-free BEGM. Sixteen hours after transfection, the cells were pretreated with 250 μM H_2_O_2 _for 2 hours and then stimulated with 10 ng/mL of IFNγ. Six hours after the IFNγ stimulation, the cells were lysed with Passive Lysis Buffer (Promega). Luciferase assays were performed using the Dual luciferase reporter assay system^® ^(Promega), and the expression of firefly and Renilla luciferase was measured with a luminometer (Lumat LB 9507^®^, Bethold, Bad Wildbad, Germany). Firefly luciferase activity was measured by adding luciferase assay reagent II (LARII, Promega), and Renilla luciferase was measured in a separate tube by adding LARII and Stop & Glo reagent (Promega).

### Knockdown of c-Jun and CREB by siRNA transfection into HBE cells

To confirm a role of c-Jun and/or CREB in H_2_O_2 _and IFNγ induced IL-32 gene regulation, c-Jun or CREB was knocked out in HBE cells using either c-Jun siRNA or CREB siRNA. HBE cells were incubated with the indicated siRNA and transfection reagent (Lipofectamine RNAiMAX, Invitrogen) for 24 hours. Then cells were stimulated with H_2_O_2 _for 2 hours and then with IFNγ for 24 hours before extraction of mRNA for evaluation of IL-32 by real time RT-PCR.

### Statistical analysis

Analysis of variance (ANOVA) with the post hoc Fisher's test was used for comparisons among groups. All analyses were performed using GraphPad Prism Ver.4 (Graphpad software, San Diego, CA, USA). Values of p < 0.05 were considered statistically significant.

## Results

### H_2_O_2 _augmented IFNγ-induced IL-32 expression in HBE cells

**Figure 1:**
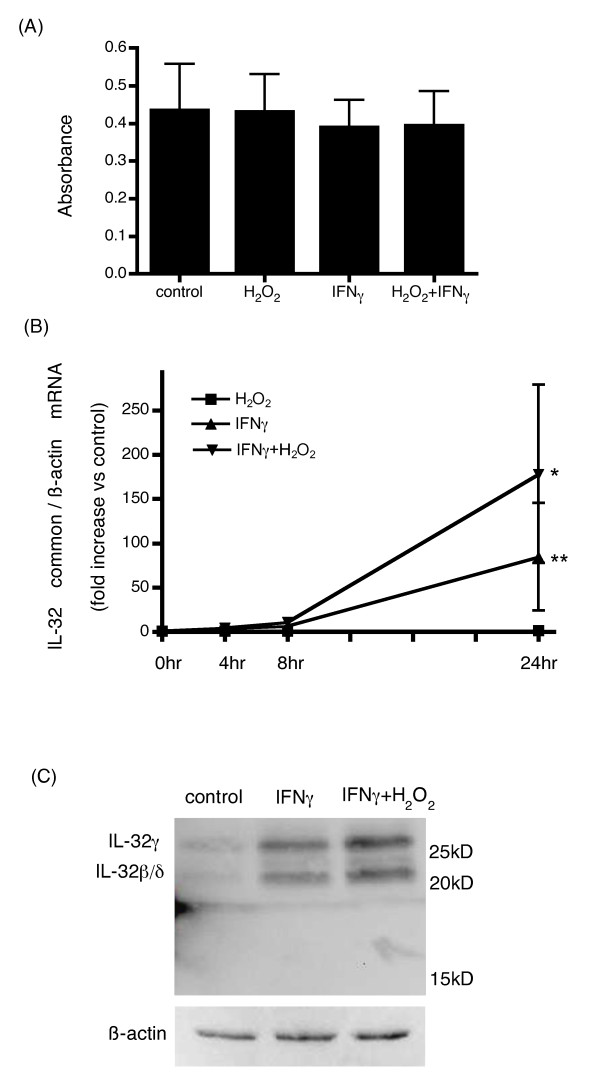
**Cell viability and IL-32 expression in IFNγ and H_2_O_2 _+ IFNγ stimulated HBE cells**. HBE cells were incubated with 10 ng/ml IFNγ or 250 μM H_2_O_2 _and 10 ng/ml IFNγ for 48 hours. The viability of the cells was examined using MTT assay (A). HBE cells were incubated with or without 2 hour-H_2_O_2 _pretreatment and then with or without IFNγ stimulation for 0, 4, 8, and 24 hours. The expression levels of IL-32 mRNA adjusted with expression levels in non-stimulated cells in each time point were determined by quantitative real-time PCR. HBE cells stimulated with H_2_O_2_, with IFNγ, and with IFNγ and H_2_O_2_, were presented in square plots, triangle plots, and inverted-triangle plots, respectively in graph (B). The bars show the means ± SE from data performed on 3 different individuals. *p < 0.01 significantly different. (C) After 48 hours of IFNγ treatment 20 μg of cell lysates were subjected to Western blotting. The 22 kDa band represents IL-32β/δ protein, and the 26 kDa band represents IL-32γ protein. The results shown are representative of 3 independent experiments.

The influence of the H_2_O_2 _concentration on the cell death of HBE cells was determined. Although treatment with 500 μM H_2_O_2 _resulted in cell death, treatment with 250 μM H_2_O_2 _did not (data not shown). The cells were confirmed to be alive at least 48 hours after stimulation with 250 μM H_2_O_2 _and/or 10 ng/ml IFNγ and the viability of the cells were confirmed by MTT assay (Figure 1A). After these preliminary experiments, we used H_2_O_2 _at a concentration of 250 μM for 2 hours prior to IFNγ stimulation in the rest of our investigations.

As shown in Figure 1B, IL-32 mRNA expression was upregulated by IFNγ stimulation time dependently. After 4 and 8 hours stimulation with IFNγ, IL-32 mRNA expression was increased by 3.6 and 6.7 times, respectively, compared with control. Furthermore, IL-32 mRNA expression was increased by 4.9 and 11.0 times after 4 and 8 hours by IFNγ with H_2_O_2_. However, synergistic upregulation by H_2_O_2 _was not significant at both times. Twenty four hours stimulation with IFNγ alone significantly upregulated IL-32 mRNA expression in HBE cells. Pretreatment with 250 μM H_2_O_2 _in addition to IFNγ synergistically upregulated IL-32 mRNA expression, although H_2_O_2 _alone did not influence its expression.

To determine the protein expression of IL-32 in HBE cells, Western blot analyses were performed using whole cell lysates from HBE cells treated with or without H_2_O_2 _and/or 48 hours of IFNγ stimulation. The antibody used for detecting IL-32 protein recognizes the 4 splice variants of IL-32, α, β, γ, and δ. However, the difference in size between IL-32β and δ was so small, it was hard to distinguish between the two in the Western blotting analyses. As shown in Figure 1C, bands appeared at 22 and 26 kDa, which represented IL-32β and/or δ, and IL-32γ, respectively. IL-32α could not be detected, suggesting that it is weakly expressed in HBE cells.

### IFNγ induced IL-32 expression was suppressed by inhibiting the JNK pathway

**Figure 2:**
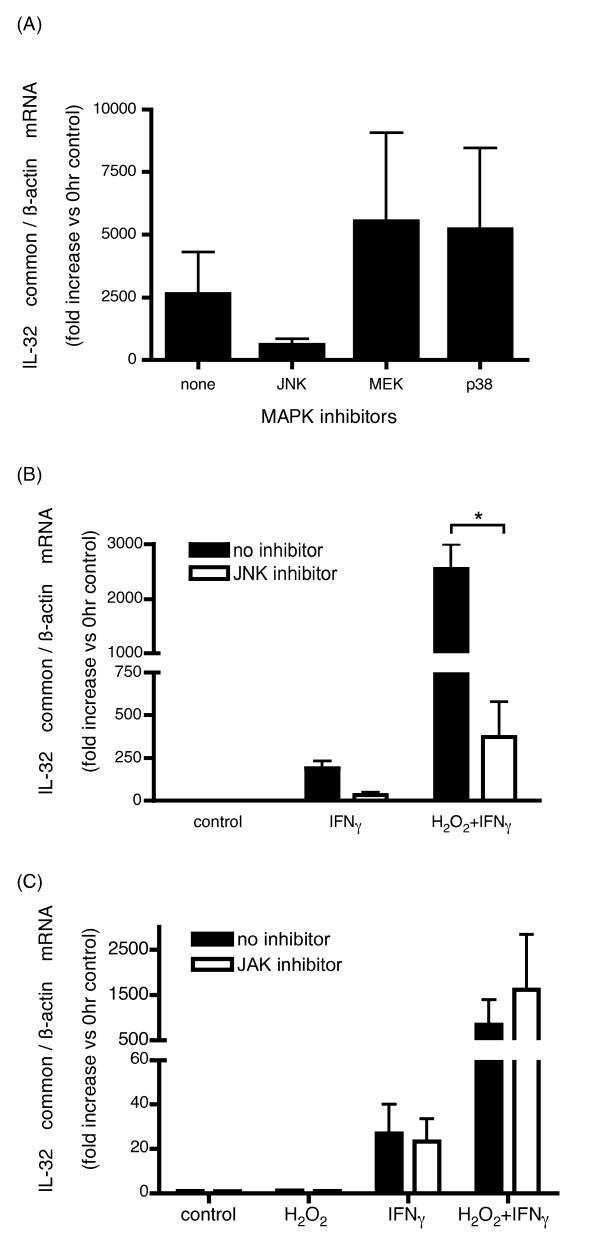
**Influence of MAPK inhibitors on H_2_O_2 _+ IFNγ induced IL-32 mRNA expression in HBE cells**. After treatment with the JNK inhibitor, the MEK inhibitor, the p38 inhibitor for 24 hours, IL-32 mRNA expression in H_2_O_2 _and IFNγ stimulated HBE cells (A) and the effect of JNK inhibitor (B) or JAK inhibitor I (C) upon IL-32 expression stimulated by IFNγ with or without H_2_O_2 _in HBE cells were examined by quantitative real-time PCR. All mRNA quantities were adjusted to the quantities at 0 hour control without stimulation. In graph (B) and (C), the closed bars represent the results of vehicle control and the open bars represent the results of JNK inhibitor (B) and JAK inhibitor (C). The bars show the means ± SE from 3 different individuals. *p < 0.05 significantly different.

To investigate which signaling pathways are responsible for regulating IL-32 in HBE cells, we examined the effects of MAPK inhibitors selective for JNK, MEK1, and p38 on H_2_O_2 _and/or IFNγ induced IL-32 mRNA expression in HBE cells (Figures 2A, B, and 2C). JNK inhibitor exerted an inhibitory effect on both IFNγ alone (Figure 2B) and IFNγ combined with H_2_O_2 _induced IL-32 mRNA expression (Figures 2A and 2B). However, neither MEK1 inhibitor nor p38 inhibitor affected the mRNA expression of IL-32 (Figure 2A).

There was no effect of JAK inhibitor I on both IFNγ alone and IFNγ combined with H_2_O_2 _induced IL-32 mRNA expression (Figure 2C).

### Stimulation of HBE cells by H_2_O_2 _followed by IFNγ promotes binding of transcription factors to the IL-32 promoter

**Figure 3:**
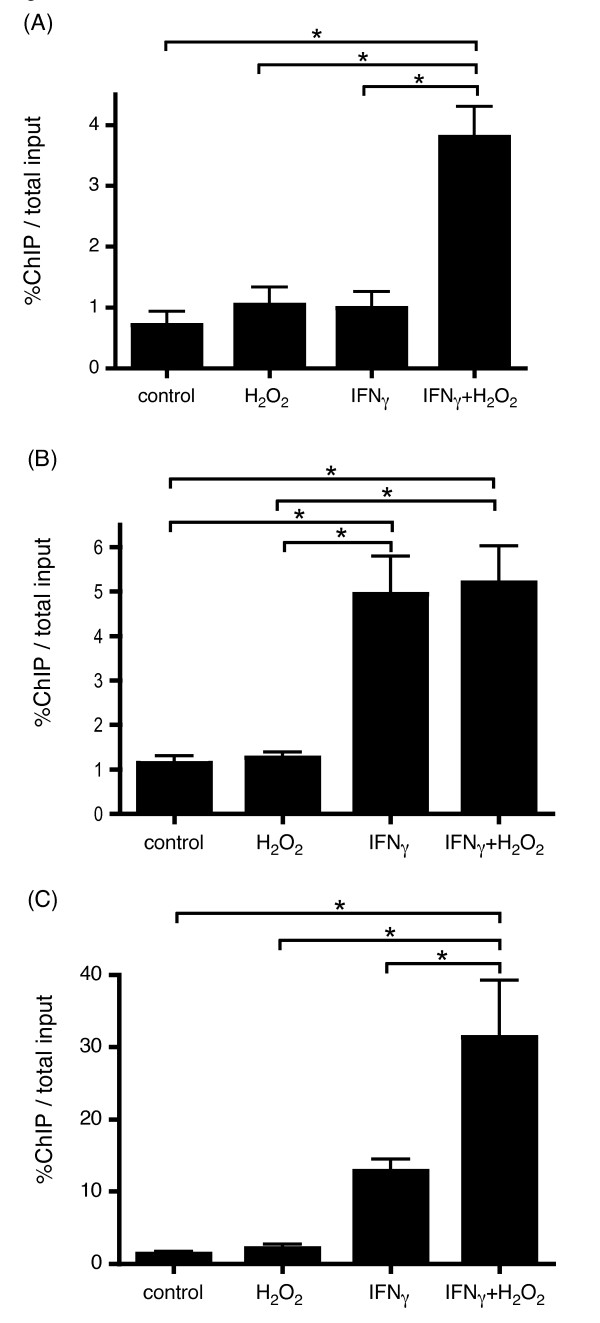
**Transcription factors that bind to the IL-32 gene promoter**. HBE cells were stimulated with H_2_O_2_, IFNγ, H_2_O_2 _+ IFNγ, or vehicle for 30 minutes. The ChIP assay was performed to identify which transcription factors bind to the IL-32 gene promoter. Binding activity was compared using quantitative real-time PCR of the IL-32 promoter in DNA from chromatin complexes immunoprecipitated by antibodies to c-Jun (A), CREB (B), and RNA polymerase II (C). The bars show the means ± SE from 3 different individuals. *p < 0.01 significantly different.

As shown in Figure 3A, c-Jun binding to the IL-32 promoter was only increased by the combined treatment of H_2_O_2 _followed by IFNγ, but not with H_2_O_2 _or IFNγ alone.

CREB binding to the IL-32 promoter was significantly increased in the IFNγ alone as well as with H_2_O_2 _followed by IFNγ (Figure 3B). As expected, H_2_O_2 _alone did not increase CREB binding to the IL-32 promoter.

RNA polymerase II binding to the IL-32 promoter was increased in the IFNγ treated cells and was further increased in the H_2_O_2_+IFNγ stimulated HBE cells (Figure 3C) These results are consistent with our results regarding the transcriptional activity of IL-32.

### Mutations in the c-Jun/CREB binding site of the IL-32 promoter largely suppressed its transcriptional activity

**Figure 4:**
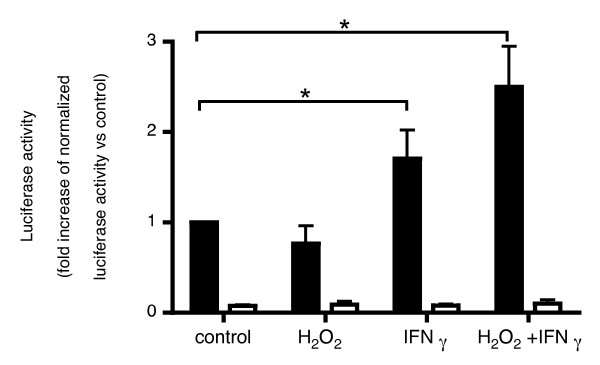
**Transcriptional activity of promoter of IL-32 gene after IFNγ or H_2_O_2 _+ IFNγ stimulation**. HBE cells were transfected with a luciferase promoter vector without mutations (pWild-Luc), closed bars, or the same vector with mutations in the c-Jun/CREB binding site (pMutant-Luc), open bars. Cells were stimulated with H_2_O_2_, IFNγ, H_2_O_2 _+ IFN, or vehicle. Six hours after indicated stimulation, the cells were lysed and luciferase activity was measured. Luciferase activity in the cells was normalized to Renilla luciferase activity. The bars represent the means ± SE from 3 different individuals. *p < 0.05 significantly different.

To confirm the roles of c-Jun and CREB in IL-32 gene transcription, which were indicated by ChIP assays, the transcriptional activity of IL-32 promoter with mutations in the c-Jun/CREB binding site was investigated. Compared to the wild-type promoter, the transcriptional activity of the mutant promoter was significantly reduced even in unstimulated HBE cells (control) and, while the wild-type promoter activity was increased by the combined stimuli of H_2_O_2 _followed by IFNγ no increase was found with promoter mutated at the c-Jun/CREB binding site, neither with H_2_O_2_, IFNγ alone, nor with the combination (Figure 4).

### Knockdown of CREB resulted in significant suppression of IL-32 induction by oxidative stress and IFNγ

**Figure 5:**
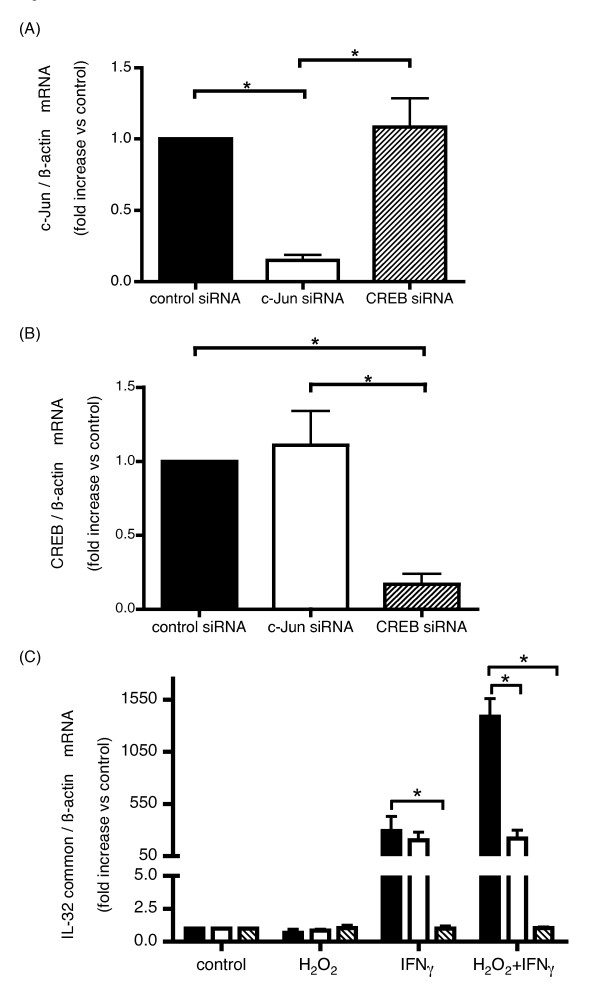
**Knockdown efficiency of c-Jun and CREB mRNA and IL-32 expression induced by IFNγ and H_2_O_2 _in HBE cells transfected with c-Jun or CREB siRNAs**. c-Jun (A) and CREB (B) mRNA expression levels examined by quantitative real time PCR in HBE cells transfected with indicated siRNAs. Expression levels of c-Jun (A) and CREB (B) by each siRNA transfection were looked by quantitative real time PCR. IL-32 expression was examined by real time PCR in HBE cells transfected with control-siRNA, closed bars, c-Jun-siRNA, open bars, or CREB-siRNA, hatched bars, respectively. Then 48 hours after transfection, cells were stimulated with H_2_O_2 _and/or IFNγ, followed by IL-32 quantitative real time PCR of RNA extracted 24 hours after the stimulation (C). The bars represent the means ± SE from 3 different individuals. *p < 0.05 significantly different.

Knock down of c-Jun and CREB mRNA expressions by each siRNA transfection in HBE cells were confirmed by real time-PCR. Both were successfully suppressed up to 10% (Figures 5A and 5B, respectively). Although knockdown of c-Jun did not influence IL-32 induction by IFNγ alone, as shown in Figure 5C, it significantly suppressed H_2_O_2 _+ IFNγ induced IL-32 expression. On the other hand, knocking down of CREB resulted in significant suppression of IL-32 expression after stimulation with IFNγ alone and also with H_2_O_2 _+ IFNγ compared with control.

## Discussion

In this study, we revealed that IFNγ upregulated IL-32 mRNA expression in HBE cells. Incubation with H_2_O_2 _alone did not upregulate its expression; however, pretreatment with H_2_O_2 _augmented IFNγ induced IL-32 mRNA and expression in HBE cells. And IL-32 induction was suppressed by JNK inhibition but not by MEK inhibition or p38 ihnibition IFNγ + H_2_O_2 _stimulated HBE cells, indicating expression induction of IL-32 by IFNγ is regulated by signal pathway involving JNK and independent of p38 or MEK. JNK inhibitor also inhibited IFNγ induced IL-32. JAK inhibitor I did not affect both on IFNγ alone and IFNγ + H_2_O_2 _induced IL-32. H_2_O_2 _alone did not increase c-Jun binding to the c-Jun binding site in the IL-32 promoter region. Binding of c-Jun was increased when HBE cells were stimulated with both H_2_O_2 _and IFNγ. On the other hand, CREB was able to bind to the IL-32 promoter after IFNγ stimulation with or without H_2_O_2_. Mutations in the c-Jun and CREB binding sites in the IL-32 promoter region inhibited the promoter activity induced by IFNγ with or without H_2_O_2_. Further, knocking down of c-Jun resulted in suppressed induction of IL-32 mRNA expression by H_2_O_2 _+ IFNγ and knocking out of CREB resulted in suppressed induction by IFNγ alone and by H_2_O_2 _+ IFNγ. This indicates that c-Jun and CREB binding to the promoter are the key mechanisms of IL-32 induction by H_2_O_2 _and IFNγ.

Several previous reports have indicated that IL-32 expression was upregulated by *Mycobacterium tuberculosis *infection or LPS in peripheral blood mononuclear cells and was also upregulated by influenza and HIV virus infection in the A549 and HEK293T human embryonic kidney cell lines, respectively. In addition, LPS and phorbol myristate acetate induced IL-32 expression in a leukemia cell line and in endothelial cells [23-26]. Recently, Li et al. reported the regulatory effect of influenza A virus upon IL-32 expression, indicating that CREB and NF-κΒ are the key molecules in the induction of IL-32 expression [27]. No previous reports have determined the regulatory effect of oxidative stress on IL-32 expression. In this study, oxidative stress; i.e., H_2_O_2 _treatment, did not affect IL-32 expression alone, but it did augment IFNγ-induced IL-32 expression in HBE cells.

Increased oxidative stress, an important aggravating factor of the disease, is persistent not only in the lungs of currently smoking COPD patients but also in patients who have achieved smoking cessation for years [10,28]. On the other hand, IFNγ is known to be a representative cytokine of CD8+ T cells and is also associated with viral infection. Even in patients with stable COPD, IFNγ levels were reported to be increased. Furthermore, viral infection is a major cause of COPD exacerbation [29,30]. Exacerbations appear to accelerate the decreasing of lung function in COPD [31]. Taken together with our findings that H_2_O_2 _did not affect IL-32 expression alone but did augment IFNγ-induced IL-32 expression, IL-32 could be induced higher in airways of COPD patients who have increased oxidative stress under the exacerbation caused by viral infection and/or an inflammatory condition in which CD8+ T cells are activated. This suggests a possible mechanism for the increased expression of IL-32 in severe COPD patients compared to mild COPD patients and non-COPD smokers, as Calabrese F et al. reported [19].

The mechanisms regulating IL-32 expression have been examined in several reports using vascular endothelial cells, synovial fibroblasts, and pancreatic cancer cell lines [13,32,33]. We investigated the mechanism regulating IL-32 expression in airway epithelial cells to clarify whether the same pathways are involved or whether characteristic features are seen according to cell type and to examine whether the blockade of certain signal pathways results in reduced expression of IL-32.

**Figure 6:**
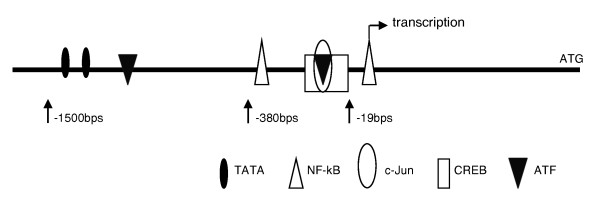
**Major transcription factor binding sites in 1500 bps upstream from transcription start site of IL-32 gene promoter**. NF-kappa B binding sites at nucleotide -9 to +5 and -361 to -352, CREB binding site at nucleotide -34 to -19, c-Jun/CREB binding site is at nucleotide -30 to -23, ATF-2 binding sites at nucleotide -33 to -20 and, -769 to -759, and TATA boxes at nucleotide -1215 to -1206 and -1502 to -1497 relative to the transcription start site at +1. These binding sites were cited by using sequence searching software TFSEARCH^(TM) ^

We have searched transcription factor binding sites existing on IL-32 promoter using sequence retrieval software TFSEARCH^(TM) ^[34], and two adjacent binding sites that could be responsible for the downstream of JNK signaling pathway are those of c-Jun and CREB, which are located between nucleotides -30 to -23 and -34 to -19, respectively, as a transcription start site at +1 (Figure 6). As shown in Figure 6, there are consensus binding sites of ATF and NFκB other than c-Jun and CREB on IL-32 promoter region. The cellular signaling pathway induced by IFNγ has been investigated in bronchial epithelial cell line (BEAS-2B) [35], which revealed that the signal was dependent on IKKB1/2 but not the NFκB pathway. In addition, IL-32 expression was not suppressed by an inhibitor of p38 which is supposed to be one of the kinases upstream of NFκB. For these reasons, NFκΒ was thought to be not associated with IL-32 expression induced by IFNγ (Figure 2A).

Further, a previous report showed that deletions of the IL-32 promoter sequence, including one of the consensus ATF binding sites, resulted in no decline in transcription activity [13]. Taking these results into consideration, we focused instead in our study on c-Jun and CREB as key molecules regulating IL-32 transcription induced by IFNγ and oxidative stress.

In HBE cells, c-Jun does not bind to the IL-32 promoter in the absence of IFNγ. Although the phosphorylation of c-Jun after H_2_O_2 _stimulation has been fully investigated in previous reports [36-38], it is unclear whether H_2_O_2 _induces or suppresses c-Jun binding to a specific gene promoter. Kumar et al. revealed that H_2_O_2 _directly suppressed AP-1 binding to the eNOS promoter [39]. Our results suggested c-Jun could be responsible for the transcription activity of IL-32 when HBE cells were stimulated by H_2_O_2 _+ IFNγ.

On the contrary, CREB bound to IL-32 promoter after IFNγ stimulation with or without H_2_O_2_. CREB activation by IFNγ has been reported previously in murine macrophages [40,41], but a larger number of reports have indicated that IFNγ has a suppressive effect on CREB activation [42,43]. There are no reports about H_2_O_2 _activating CREB binding to gene promoters. In our study, CREB was bound to the IL-32 promoter after IFNγ treatment but not after treatment with H_2_O_2_ alone. The activation and binding of CREB to the IL-32 promoter by IFNγ is one possible mechanism of IL-32 gene regulation in HBE cells and another is that IFNγ indirectly affects CREB activation through coactivators (e.g., CBP/p300) that are induced and activated by IFNγ [44] or other transcription factors including STAT-1 [45,46], which is the main downstream effector of IFNγ stimulation. IFNγ is known to exert its effect through not only JAK/STAT signal pathway but also through MAPK signaling pathway [47,48]. Some reports indicated presence of MAPK signal transduction by IFNγ which is not suppressed by inhibiting JAK/STAT [49], and Kim HA et al found IFNγ signal exerted by activation and upregulation of CREB but is not influenced by knocking out of STAT [40]. These research findings support our results that IL-32 expression regulation by IFNγ can be dependent on JNK (and its downstream c-Jun and CREB) but be independent of JAK/STAT (Figure 2C). Although no binding sites for STAT or interferon regulatory factor (IRF) were detected, but CREB binding site was detected at least within 1500 bps from transcription start site of IL-32 promoter, further investigation with the full length promoter will be needed to confirm whether the JAK/STAT pathways are involved or not.

As shown in Figure 4, mutations in the c-Jun/CREB binding sites resulted in markedly reduced transcription of IL-32, even after H_2_O_2 _and/or IFNγ stimulation. This is consistent with a previous report investigating the regulatory mechanism of IL-32 transcription in endothelial cells by Kobayashi et al. [13]. They demonstrated that the deletion of the IL-32 promoter between nucleotides 26 and 100 upstream of the transcription initiation site, identical to the region containing the CREB and c-Jun binding sequences, led to significantly suppressed transcription activity. Further, knocking down of CREB by siRNA transfection resulted in a significant suppression of the IL-32 expression that was induced by both IFNγ alone and IFNγ + H_2_O_2 _down to the baseline control levels. CREB is a key transcription factor for IL-32 transcription in HBE cells. This finding is also compatible with the report by Li et al. showing that mutations in the binding site for CREB or CREB knockdown resulted in the significant suppression of influenza A virus-induced IL-32 transcription [27]. In addition, decreased expression of c-Jun by c-Jun siRNA resulted the significant suppression of IL-32 induction by IFNγ + H_2_O_2_, though IL-32 induction by H_2_O_2 _or IFNγ alone was not affected (Figure 5C). These findings suggested synergistic effect of c-Jun inducing IL-32 expression by IFNγ under oxidative stress.

Although p38 is suggested to be one of MAPKs located upstream of AP-1 (including c-Jun), we found no effect of p38 inhibitor on IL-32 induction by IFNγ in HBE cells. This could be explained by number of reports showing that signal transduction induced by cytokines such as IFNγ or oxidizing substance that involve AP-1 was dependent of JNK but was independent of p38 in HBE cells and macrophages [50,51]

Putting the results of our experiments together, we suggest that IFNγ induced CREB binding to the IL-32 promoter, which was followed by an increase in the transcription of IL-32, and an additive effect of c-Jun binding to the IL-32 promoter by H_2_O_2 _resulted in a further acceleration of IL-32 transcription. How IFNγ affects c-Jun binding to the promoter has not been clarified. One possibility is that it involves a coactivator, e.g. CBP/p300, which is known to bind to AP-1 similar to c-Jun and promotes transcriptional activity [52]. CBP/p300 could be activated by IFNγ [49], and CREB could form a heterodimer with c-Jun [53], which may explain the necessity of IFNγ for the binding of c-Jun to the IL-32 promoter during H_2_O_2 _stimulation. In addition, both activated c-Jun and CREB binding to the IL-32 promoter are necessary to induce significant transcription of IL-32 in IFNγ and H_2_O_2 _stimulated HBE cells.

The role of increased IL-32 expression in airway epithelial cells in the pathogenesis of COPD has not been clarified, and furthermore, it is still unclear whether suppressing its expression is beneficial with regards to preventing disease progression or improving the symptoms of the disease. IL-32 has several roles including inducing the expression of inflammatory cytokines and adhesion molecules in T-lymphocytes, monocytes, macrophages, and epithelial cells and promoting monocyte differentiation into macrophages, which alters the responses of inflammatory cells against infection [54,55]. Also, proteinase-3, which is activated by neutrophilic inflammation, cleaves IL-32 into its highly activated form [17,56]. Thus, increased IL-32 expression may modify airway inflammation in COPD. Further investigations to identify the roles of IL-32 in COPD are necessary.

As IL-32 is expressed in several types of cells, the mechanism of IL-32 gene regulation suggested in this study may not only be applicable to the pathogenesis of COPD but also those of other inflammatory diseases associated with oxidative stress. Further studies are needed.

## Abbreviations

IL-32: interleukin-32; COPD: chronic obstructive pulmonary disease; HBE: human bronchial epithelial cell; ChIP: chromatin immunoprecipitation; CCL: CC chemokine ligand; CXCL: CXC chemokine ligand; TSLP: thymic stromal lymphopoietin; GM-CSF: granulocyte macrophage colony stimulating factor; LPS: lipopolysaccharide; FEV_1_: forced expiratory volume in one second; DMSO: dimethyl sulfoxide; PCR: polymerase chain reaction; RT-PCR: Reverse Transcription -polymerase chain reaction; eNOS: endothelial nitric oxide synthase.

## Competing interests

The authors declare that they have no competing interests.

## Authors' contributions

MK and EO performed the whole experiments. DK, AH, TT, NT and SM contributed in collecting clinical data and samples. HS and HD contributed in providing surgery samples. EO designed the study. DK, AH, TT, NT, YH, TH, SM, and MM advised the design of the study and participated in the analysis of the data. All authors read and approved the final manuscript.
